# Organic Electroluminescent Materials Possessing Intra- and Intermolecular Hydrogen Bond Interactions: A Mini-Review

**DOI:** 10.3389/fchem.2022.954419

**Published:** 2022-07-22

**Authors:** Xinyong Liu, Jingwei Li, Xu Qiu, Yuyu Pan

**Affiliations:** ^1^ College of Materials Science and Engineering, Shandong University of Science and Technology, Qingdao, China; ^2^ School of Petrochemical Engineering, Shenyang University of Technology, Liaoyang, China

**Keywords:** hydrogen bond, intra- and intermolecular interaction, charge transfer, organic emitter, optoelectronic property

## Abstract

Organic light-emitting diodes (OLEDs) have become the predominant technology in display applications because of their superior light weight, flexibility, power conservation, and environmental friendliness, among other reasons. The device’s performance is determined by the intrinsic properties of organic emitters. The aggregation structure of emitters, in particular, is crucial for color purity and efficiency. Intra- and intermolecular interactions, such as hydrogen bonds (H-bonds), can reduce structural vibrations and torsions, which affect the stability of emitting layer films and optoelectronic properties of emitting materials. Hence, by regulating the H-bond interaction, the desired properties could be obtained. This mini-review focuses on the influence of intra- and intermolecular H-bond interactions on the optoelectronic properties of high-performance emitters.

## Introduction

As stated in the pioneering work of Tang and VanSlyke et al., organic light-emitting diodes (OLEDs) have attracted considerable scientific and industrial interests (Tang and VanSlyke, 1987). Extensive research has been conducted to promote OLEDs in commercial applications as flat panel displays and lighting sources due to their lightweight, flexibility, power saving, and environmental friendliness (Chen and Xu, 2021; Zhang et al., 2021; Zhu et al., 2021). Red, green, and blue (RGB) emitters with nearly comparable stability, efficiency, and color purity are necessary to fabricate full-color flat panel displays. Therefore, developing emitters with good comprehensive performance is crucial for the new generation of full-color flat panel display applications.

Organic emitters are one of the indispensable parts of OLEDs; not only the properties of the monomolecular state but also those of the aggregated state can influence device performance. The aggregation structure of emitters is crucial for device color purity and electroluminescence (EL) efficiency (Guo et al., 2017; Han et al., 2020). Intra- and intermolecular interactions, such as hydrogen bonds (H-bonds), could have a vital effect on the stability of emitting layer films and optoelectronic properties of emitting materials. The desired optoelectronic properties could be obtained by regulating the H-bond interactions. Hence, in this mini-review, we focus on the organic emitting materials from the influence of intra- and intermolecular H-bond interactions on optoelectronic properties.

## Intra- and Intermolecular H-bond Interactions in Different Emitting Materials

The intra- and intermolecular interactions in optoelectronic materials can influence their arrangement and aggregation behaviors, which could further influence, for example, carrier mobility, color purity, and efficiency. This section mainly discusses the influence of intra- and intermolecular H-bond interactions on the optoelectronic properties of RGB organic emitting materials.

### H-bond Interactions in Organic Blue Emitters

An efficient deep-blue emitter can lower the power consumption, increase the color gamut of full-color OLEDs, and create other visible emissions and white light through the energy transfer processes (Lv et al., 2021; Xu et al., 2021). However, deep-blue emitters have a naturally broad bandgap, leading to a significant charge injection barrier and unbalanced charge injection and transportation in the device (Xue et al., 2017; Zhang et al., 2020; Zhang et al., 2022). Therefore, there is an essential and significant need to develop deep-blue emitters with high EL efficiency and narrow-band emission.

**FIGURE 1:**
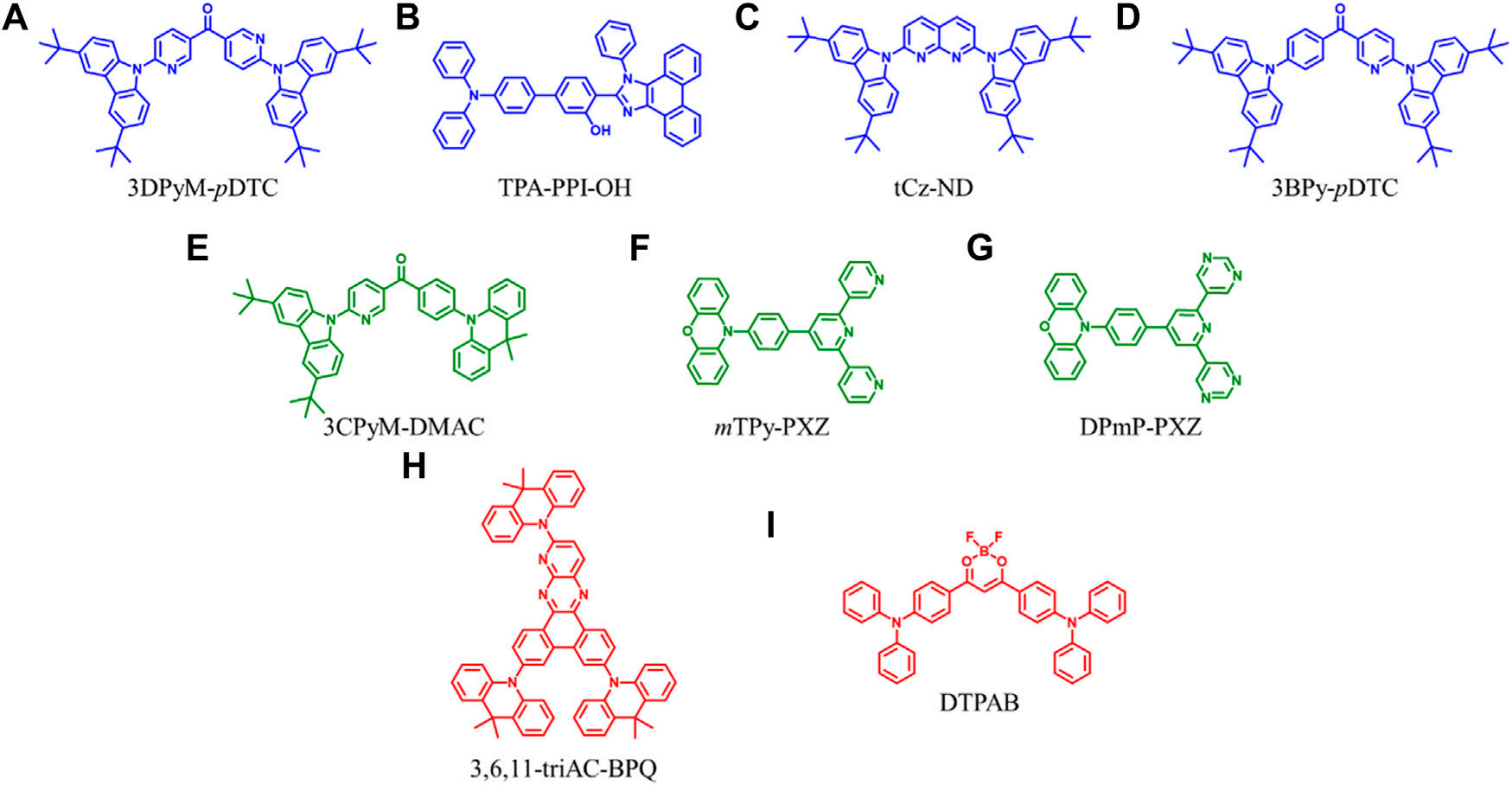
Chemical structures of the OLED materials possessing intra/intermolecular H-bond interactions.

**FIGURE 2:**
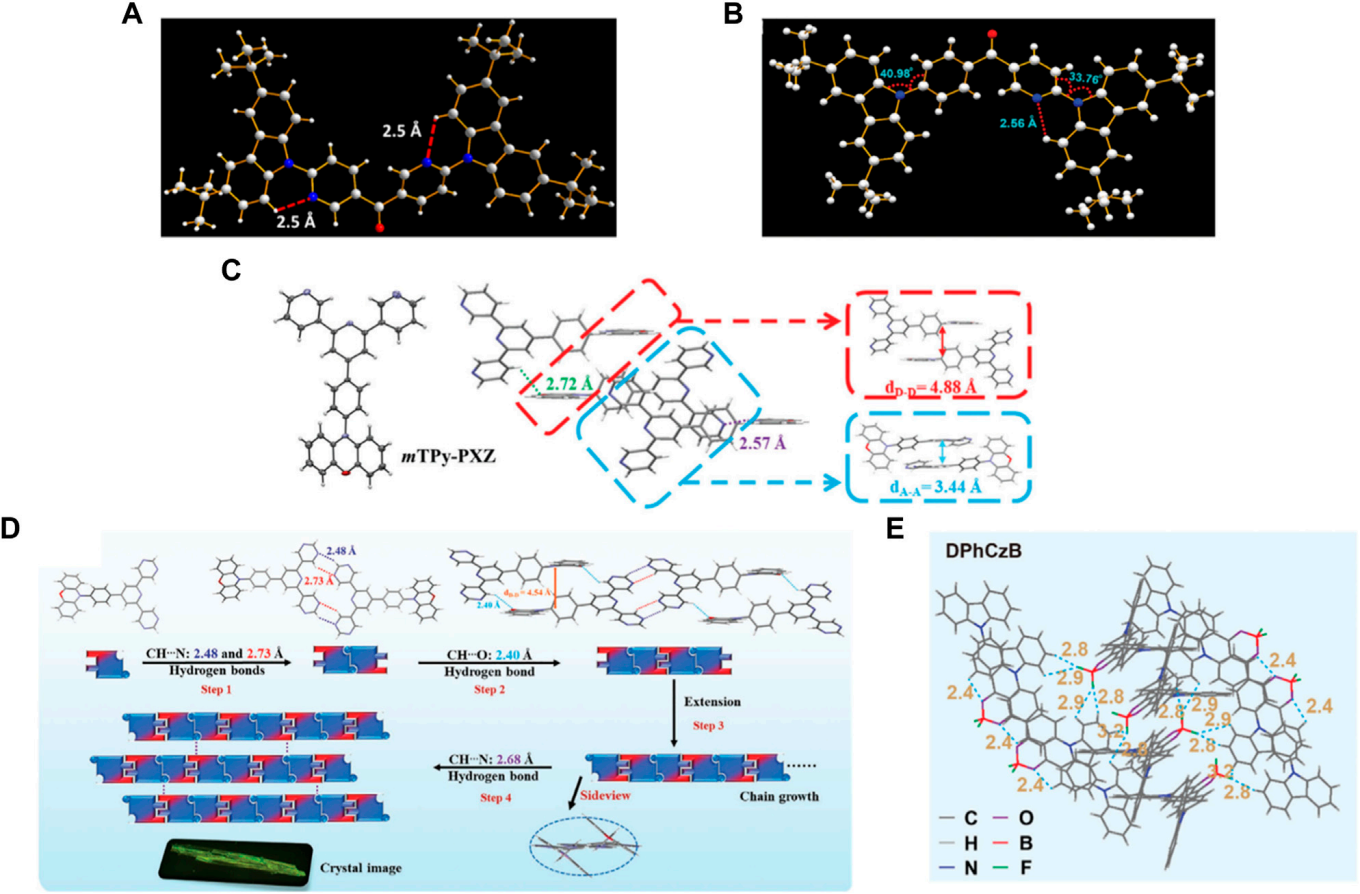
The H-bond interactions in different OLED materials. **(A)** Crystal structure of 3DPyM-*p*DTC. **(B)** The molecular structure and packing diagram of 3BPy-*p*DTC obtained from single-crystal X-ray diffraction analysis displaying the intramolecular H-bonding interaction and the donor-acceptor dihedral angles. **(C)** Thermal ellipsoid drawings at the 50% probability level and intermolecular geometries of *m*TPy-PXZ in the single crystals were determined by X-ray analysis. **(D)** Intermolecular packing geometries of DPmP-PXZ in the single crystal determined by X-ray analysis. **(E)** Molecular packing and inter- and intramolecular interactions in the DPhCzB crystal.

To simultaneously enhance color purity, out-coupling efficiency, and internal quantum efficiency of OLEDs, two isomers, 2DPyM-*m*DTC and 3DPyM-*p*DTC (Figure 1A), have been designed by Cheng et al. The crystal structure of 3DPyM-*p*DTC showed that the intramolecular H-bonding between the two pyridine nitrogen atoms and the proximal C−H-bonds of the tert-butylcarbazole groups with a C‒H···N of 2.5 Å was found (Figure 2A). The presence of C‒H···N hydrogen bonding should limit rotation between the donor and acceptor groups in the molecule and increase the photoluminescence quantum yield (PLQY) in the solid state. The device based on 3DPyM-*p*DTC, with a nearly planar structure, shows a very high PLQY of 98%, EQE of 31%, and corresponding blue emission with full width at half-maximum (FWHM) of 62 nm and CIE of (0.14, 0.18) compared with 2DPyM-*m*DTC (Rajamalli et al., 2017).

Ma et al. reported TPA-PPI-OH (Figure 1B), a deep-blue fluorescent emitter with phenol group as a π-bridge (Qiu et al., 2019). The endowed intra- and intermolecular H-bonds interactions proved beneficial in suppressing the structural vibrations and thereby caused a narrower FWHM PL emission of TPA-PPI-OH. A non-doped OLED device based on TPA-PPI-OH exhibited highly efficient EL performance and achieved an EQE of 7.37% with narrow emission (FWHM: 58 nm). Kazlauskas et al. exploited carbazole–naphthyridine (donor-acceptor) based blue-emitting compounds, which were designed using both the H-bonding and sterically controlled charge-transfer interactions between D and A units. None methyl-substituted naphthyridine (tCz-ND, Figure 1C) exhibited deep-blue (λ_max_ < 460 nm) and narrow-band EL (FWHM = 66 nm), whereas the more twisted methyl-substituted compound (MetCz-ND) expressed broader band (FWHM >80 nm) sky-blue (λ_max_ ≈ 480 nm) emission. (Kreiza et al., 2020). Recently, Rajamalli et al. demonstrated the role of the donor substitution position in a thermally activated delayed fluorescence (TADF) emitter to achieve deep-blue emission with improved color purity without reducing the device performance. A novel 3BPy-*p*DTC (Figure 1D) was synthesized, where two tert-butyl carbazolyl (DTC) donors linearly connected at the para position of the benzoyl pyridine (3BPy) acceptor core. 3BPy-*p*DTC shows higher color purity in deep-blue emission than the meta-substituted counterpart (3BPy-*m*DTC) due to the locked geometry *via* intramolecular H-bonding (Figure 2B) (Sk et al., 2022).

### H-Bond Interactions in Organic Green Emitters

TADF materials are urgently needed for fabricating OLEDs because of the high exciton usage efficiency and metal-free molecular frameworks (Liu et al., 2018). The narrowing singlet-triplet splitting energy (ΔE_st_) is important for the up-conversion process from triplet to singlet excitons in devices, resulting in theoretically high internal quantum efficiencies. Qi et al. discovered that the existence of intramolecular hydrogen bonding is conducive to diminish the energy difference (ΔE_st_) between a singlet and a triplet, suppressing nonradiative decay and increasing the luminescence efficiency (Ma et al., 2020). They found that, for the crystals of CBM-PXZ and 3CPyM-PXZ, multiple H-bonds of C=O···H with the distances of 2.57–3.57 Å can be observed, which were conducive to locking the movement within molecules and rigidifying the geometric structures of molecules. Therefore, the nonradiative decay process can be suppressed, and luminescence efficiency will be enhanced in the solid state. Therefore, solution-processed non-doped OLEDs adopted 3CPyM-DMAC (Figure 1E) as an emitter exhibiting a maximum CE and EQE of 33 cd A^−1^ and 11.4%.

Due to quenching caused by intermolecular triplet contact, non-doped OLEDs always result in significant efficiency roll-off. Zhang et al. reported a green fluorescent material of *m*TPy-PXZ (Figure 1F), revealing a novel strategy of tuning intermolecular H-bonds for high-performance non-doped electroluminescence (Shi et al., 2020). Suitable intermolecular H-bond interaction enables the 3D supramolecular framework formation (Figure 2C), which limits the nonradiative process and suppresses the triplet exciton quenching caused by π–π stacking of triplets but also favors the horizontal molecular orientations, especially in their non-doped states. The non-doped OLED based on the *m*TPy-PXZ with such suitable intermolecular H-bonds exhibits the state-of-the-art performance with maximum EQE of up to 23.6% with only 7.2% roll-off at 1,000 cd m^−2^. Recently, they designed a new TADF emitter, DPmP-PXZ (Figure 1G), composed of 2,6-di (pyrimidin-5-yl) pyridine (DPmP) as electron-acceptor and phenoxazine (PXZ) as electron-donor (Shi et al., 2021). Further intermolecular hydrogen bonding between the DPmP and PXZ groups favors the formation of extended linear chains of molecules instead of 3D frameworks (Figure 2D). It is further shown that the 1D structure would help separate electron-rich PXZ cores in neighboring molecules. This leads to suppression of exciton annihilation between molecules, and the extended 1D chain structure improves the carrier mobility balance and optical out-coupling. The non-doped device based on DPmP-PXZ realized an excellent maximum EQE of 21.8% with little efficiency roll-off. These findings contribute to a better understanding of the role of hydrogen bonding in molecular packing and expand the possibilities for using varied hydrogen bonding to regulate molecular packing in non-doped systems.

### H-Bond Interactions in Organic Red Emitters

The design of high-performance red emitters remains a great challenge due to their small energy bandgaps with severe nonradiative decay for low luminous efficiency. Introducing rigid and fused coplanar molecular structure to suppress the vibrational relaxation and show a horizontal molecular orientation in the film, enhancing the luminescence efficiency of organic red emitters, is an effective technique. Tang et al. fabricated an emitter, 3,6,11-triAC-BPQ (Figure 1H), containing a rigid planar dibenzo[*f,h*]pyrido[2,3-*b*]quinoxaline (BPQ) core and three 9,9-dimethyl-9,10-dihydroacridine (Ac) donors (Xie et al., 2020). They found that a 3,6,11-triAC-BPQ intramolecular H-bond refined the dihedral angle, which can hybrid the local and charge transfer excited state. Finally, a device with 3,6,11-triAC-BPQ as an emitter exhibited a high EQE of 22.0%.

Introducing rigid and fused moieties is an effective way to enhance the red emitters’ luminescence. Still, the solubility is significantly reduced, inevitably prohibiting their applications in solution-processed OLEDs. Hence, Chen et al. proposed an intermolecular locking strategy to improve the solution processibility and photoluminescence efficiency of red emitters using a highly soluble flexible difluoroboron β-diketonate unit with exposed and easily reachable fluorines that can form H-bonds in the solid state to induce strong intermolecular locking for high luminescent efficiency (Jin et al., 2021). Due to the exposed difluoroboron β-diketonate group with multiple fluorine and oxygen atoms in forming hydrogen bonds, abundant intra- and intermolecular interactions with short distances can be observed with strong intermolecular hydrogen bonds of C–H‧‧‧F and C–H‧‧‧O in the single-crystal structure analyses of DPhCzB (Figure 2E). Hence, the solution-processed OLED based on DTPAB (Figure 1I) exhibits exceptional high performance, with a maximum EQE of 8.2%. These results demonstrated that the intermolecular locking strategy by directly addressing the internal conflicts between solubility and luminescent efficiency provides important clues in developing highly efficient and solution-processable red emitters for high-performance OLEDs.

## Conclusion

One of the most promising technologies for future lighting and flat panel display applications is highly efficient OLEDs. In this mini-review, we have mainly discussed the RGB emitting materials possessing intra- and intermolecular H-bonds interactions that potentially affect optoelectronic performance. There are mainly two aspects ascribed to the interactions based on exploiting the intra- and intermolecular H-bonds interactions and analyzing the performance of different materials: 1) restricting the rotation between different donor/acceptor moieties and inhibiting the vibrational coupling of excited states, which could obtain high luminous efficiency and color purity; 2) the multiple H-bonds interactions could further enhance horizontal orientation in amorphous organic semiconductor films and significantly increase hole and electron mobilities, which is beneficial for efficiency stability with negligible roll-off. Although the H-bond interaction is weaker than a covalent interaction, it is critical in promoting the development of high-performance OLEDs. Optoelectronic materials with hydrogen bonding interactions will, predictably, attract increasing interest and attention in the future.
